# Refining Cultural Adaptations of a Behavioral Intervention for Latino Caregivers of People Living With Dementia: Qualitative Interview Study in Washington State

**DOI:** 10.2196/53671

**Published:** 2024-01-11

**Authors:** Celeste N Garcia, Miriana C Duran, Magaly Ramirez

**Affiliations:** 1 Department of Health Systems and Population Health University of Washington Seattle, WA United States

**Keywords:** caregivers, caregiver, caregiving, carer, carers, STAR-C, STAR caregiver, internet, web-based, online, educational, education, family care, family, families, informal care, adaptation, adaptations, cultural, culturally, module, modules, training, Hispanic, Hispanics, Spanish, Latin, Latina, Latinas, Latinos, Latinx, Latino, dementia, qualitative research, Alzheimer disease, qualitative, Alzheimer, experience, experiences, attitude, attitudes, opinion, perception, perceptions, perspective, perspectives, aging, older adults, old age, mental health, neuro, ageing, geriatrics, gerontology, geriatric, interview, eHealth, digital health, alzheimers, memory, memory loss, care giving, Hispanic or Latino, mobile phone

## Abstract

**Background:**

In the United States, Latino caregivers of individuals with dementia face unique challenges and an elevated risk of adverse health outcomes. Despite the increasing prevalence of Alzheimer disease and related dementias among Latino adults, few evidence-based interventions are tailored to their cultural context. To address this gap, we examined the cultural adaptations required for the STAR caregivers (STAR-C) virtual intervention, an evidence-based intervention that educates family caregivers to manage behavioral and psychological symptoms of dementia. While STAR-C has shown effectiveness, neither the original in-person nor the virtual intervention considered the distinct experiences of Latino caregivers, who often bring culturally significant values into caregiving interactions.

**Objective:**

This study’s objective was to test and refine the preliminary cultural adaptations of the STAR-C web-based training modules for Latino caregivers of people living with dementia.

**Methods:**

Through qualitative interviews with 15 Latino caregivers in Washington State, we identified key adaptations to enhance the cultural relevance of the web-based training modules.

**Results:**

The interviews highlighted 4 main themes for adaptation: the delivery of the STAR-C web-based training modules, comprehensive dementia education, simplified problem-solving strategies, and prioritizing caregiver well-being.

**Conclusions:**

This study’s findings informed the development of culturally adapted STAR-C web-based training modules that aim to provide tailored support to Latino caregivers. While further research is needed to assess the efficacy of these adaptations, our work contributes to bridging the gap in dementia caregiving for Latino families, potentially reducing health disparities and enhancing health care services for this population.

## Introduction

Latino caregivers of people living with dementia are at an increased risk of experiencing adverse health impacts due to caregiving, yet few evidence-based interventions have been developed to support Latino families [1]. In the United States, Latino adults are 1.5 times more likely to develop Alzheimer disease and related dementias (ADRD) compared to non-Latino White adults [2]. The disparity is due in large part to the health conditions (eg, cardiovascular disease, diabetes, high blood pressure, and obesity) and socioeconomic factors (eg, chronic exposure to economic and social adversity, lower levels and quality of education, and discrimination) that are more prevalent in Latino populations and are associated with cognitive decline [2,3]. The number of Latino people living with dementia is expected to increase to 3.5 million by 2060, leading to a rise in Latino adults caring for family members with ADRD [4]. Although evidence-based caregiver interventions exist, they often fall short in meeting the unique cultural needs of Latino families [1,5,6]. There is an urgent need to develop culturally appropriate evidence-based interventions that address the unique challenges faced by Latino caregivers of people living with dementia and consider the sociocultural context in which they provide care.

STAR caregivers (STAR-C) is an in-home intervention that involves training health professionals to teach family caregivers strategies to manage behavioral and psychological symptoms of dementia (BPSD) [7,8]. Caregivers learn to monitor symptoms, identify possible environmental or interpersonal triggers, and develop effective responses. They also learn strategies for communicating with people living with dementia in a way that supports positive affect and prevents or minimizes problems, increasing pleasant events to improve mood, and improving the support caregivers receive from informal and formal networks. STAR-C is demonstrated to reduce the frequency and severity of BPSD, as well as improve burden, depression, and reactivity to symptoms in caregivers [7]. Recently, STAR-C was reconfigured as a virtual intervention to facilitate large-scale implementation in clinical settings. The virtual intervention, coined STAR-C Virtual Training and Follow-up is being tested in an ongoing trial at Kaiser Permanente Washington [9,10]. For 6-8 weeks, caregivers complete 6 web-based training modules asynchronously and have six 30-minute weekly telephone check-ins with a coach (ie, master’s-level social worker or mental health counselor). In addition, support from coaches is provided, as needed, via secure messaging in the Kaiser Permanente Washington patient portal for up to 6 months.

The STAR-C virtual intervention was timely given the COVID-19 pandemic, which shed light on the urgent need for digital health strategies that offer support virtually [11]. Many social and health care services for older adults and their family caregivers shifted from in-person to digital platforms to expand reach during the pandemic [12]. Neither the in-person or virtual STAR-C interventions, however, were developed with explicit consideration of the experience of Latinos providing care to a family member living with ADRD. Caregiver interventions for Latinos need cultural adaptations because cultural values and beliefs, such as *familismo* (dedication and commitment to family) and *respeto* (respect) play a pivotal role in shaping caregiving interactions with people living with dementia, experiences, and perceptions of support [13]. The goal of performing cultural adaptations to evidence-based interventions such as STAR-C is to promote more favorable experiences with the intervention and alleviate the health disparities associated with dementia caregiving among vulnerable populations.

To address the gap in the lack of culturally appropriate evidence-based interventions for Latino caregivers, we sought to culturally adapt the content of the web-based training modules of the STAR-C virtual intervention for Latino caregivers. In our previous study, we identified what cultural adaptations to the STAR-C web-based training modules are needed for Latino caregivers and we designed preliminary adaptations [14]. Preliminary adaptations included expanding the content of the web-based training modules to improve understanding of dementia; revising language that was viewed as stigmatizing, offensive, or culturally inappropriate; and adding cultural examples to reflect the range of family involvement in caring for people living with dementia and multigenerational living [14]. It is unknown, however, whether these modifications sufficiently align with the intended goals of cultural adaptation [15]. In addition, it is unknown whether there are additional opportunities to further enhance the cultural relevance of the STAR-C web-based training modules for Latino families. Therefore, the objective of this study was to test and refine the preliminary cultural adaptations of the STAR-C web-based training modules for Latino caregivers of people living with dementia.

## Methods

### Ethical Considerations

This study was granted approval by the institutional review board at the University of Washington (STUDY00009534). Participants in this study gave their verbal or written consent for their involvement. All the data we collected from participants was labeled with a unique study identification number and not the participants’ name or any other information that could identify participants. The contact information of participants was kept in a password-protected file and computer. All data collected from participants were kept confidential and accessible only by our study team. We did not use participants’ names in reports of study findings, REDCap (Research Electronic Data Capture; Vanderbilt University) surveys, or audio recordings of interviews. Instead, we labeled everything with this study’s identification number. We destroyed data that identified participants when we finished recruitment.

### Overview of This Study’s Design

**Figure 1:**
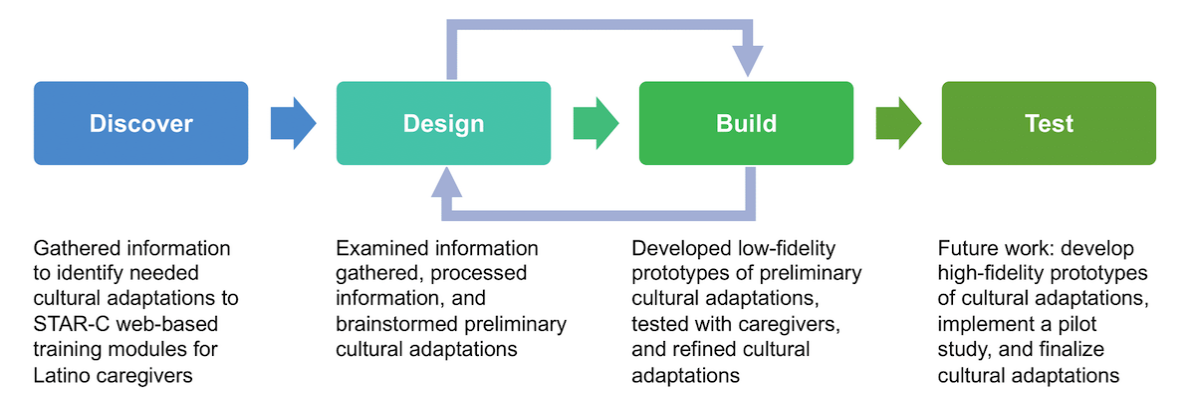
The cultural adaptation of STAR-C web-based training modules within the context of the Discover, Design + Build, and Test Framework. STAR-C: STAR caregiver.

Figure 1 [16] illustrates our approach to the cultural adaptation of the STAR-C web-based training modules within the context of the Discover, Design + Build, Test framework. In our previous study, we conducted a qualitative study to gather information about needed cultural adaptations to the web-based training modules for Latino caregivers (the “Discover” phase) [14]. We then used the findings from the qualitative study to brainstorm ideas for preliminary cultural adaptations (the “Design” phase). In this study, we developed low-fidelity prototypes of the culturally adapted STAR-C web-based training modules and tested the prototypes with Latino caregivers (the “Build” phase).

### Participant Selection

We carried out semistructured qualitative interviews in Washington State with 15 Latino caregivers of people living with dementia who spoke Spanish or English. Caregivers were considered eligible if they self-identified as Hispanic or Latino, were aged 21 years or older, were a child, spouse, or partner, or close friend of someone diagnosed with dementia, lived with the diagnosed individual or within a 5-mile radius, and provided a minimum of 8 hours of weekly care. Our recruitment strategy involved 3 approaches. Initially, we identified potential participants through the electronic health record system at University of Washington Medicine. Additionally, we circulated flyers in both Spanish and English across various locations, including a UW Medicine specialty clinic, a primary care practice-based research network, the Alzheimer Association Washington State Chapter, and local *tiendas* in Latino communities. Lastly, we used media platforms like local Spanish and English radio stations and newspapers to discuss the impact of dementia on the Latino population and publicize this study.

A member of this study’s team screened for eligibility the individuals who showed interest in joining this study and arranged interviews for those who met the eligibility requirements. Caregivers received a compensation of US $45 for taking part in this study.

### Description of the Low-Fidelity Prototypes

In the STAR-C Virtual Training and Follow-up intervention, caregivers receive 1 web-based training module per week for a period of 6-8 weeks. Textbox 1 describes the topics of the web-based training modules. The core components of the intervention include dementia education, strategies for effective communication, Activators-Behaviors-Consequences (ABC) problem-solving, pleasant events, and caregiver support.

**Topics of STAR caregivers (STAR-C) web-based training module.**

**Week 1:** Understanding dementia, realistic expectations about behavioral treatments for behavioral and psychological symptoms of dementia, and strategies for effective communication.

**Week 2:** Activator, Behavior, Consequence (ABC) approach to problem-solving, including rationale and development of an ABC plan for target behaviors that caregivers identify.

**Week 3:** Review of ABC plan (revise if needed).

**Week 4:** Pleasant events and managing negative thinking.

**Week 5:** Review of ABC plan and pleasant events schedule (revise if needed).

**Week 6:** Caregiver support strategies for coping with caregiving and maintaining gains.

**Figure 2:**
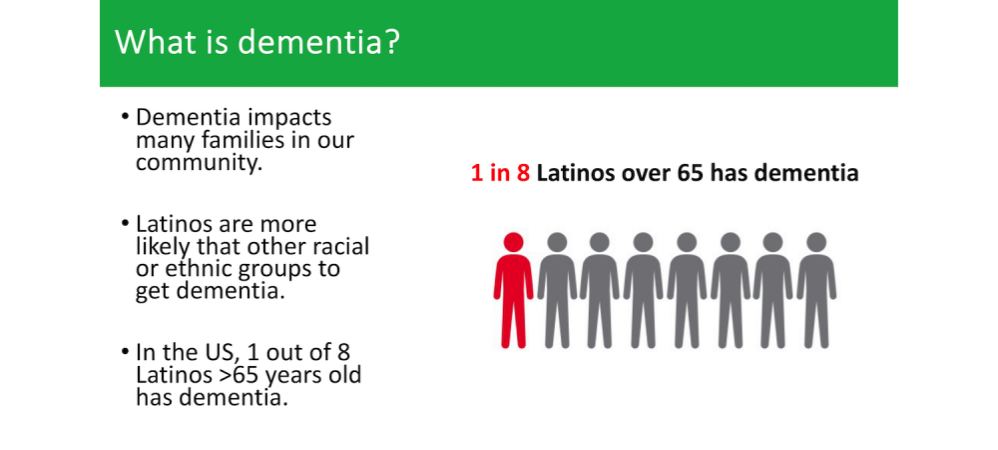
Culturally adapted STAR-C web-based training module—understanding Alzheimer and related dementias, with a focus on disproportionate impact on Latinos. STAR-C: STAR caregiver.

**Figure 3:**
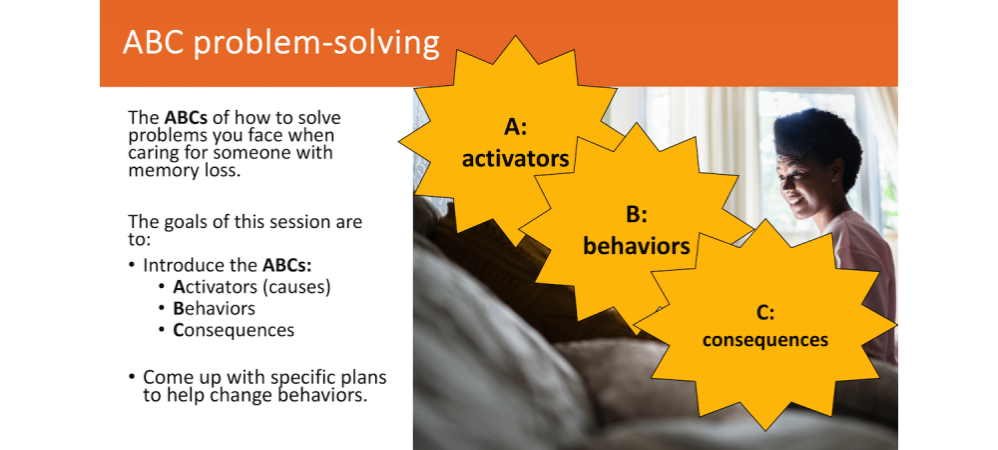
Culturally adapted STAR-C web-based training module—ABC acronym. ABC: Activators-Behaviors-Consequences; STAR-C: STAR caregiver.

We created 3 low-fidelity prototypes of the culturally adapted STAR-C web-based training modules in both English and Spanish for testing among study participants. The low-fidelity prototypes were in the form of videos of recorded presentations with images, text, and voice-over. The videos featured short excerpts from various STAR-C web-based training modules. The first video consisted of educating Latino family caregivers on dementia and problem-solving using the ABC approach. This video explained the STAR-C program, dementia, its causes, walked through each step of the ABC approach for problem-solving in dementia, and concluded with a caregiving example of the ABC approach. The second video featured some modifications, including the addition of information about dementia stages, common behaviors exhibited by people living with dementia, and importance of self-care. We also enhanced the visual aesthetics and design of the lessons to make them more visually appealing and added humor. In the third video, we maintained the core content from the previous versions but incorporated interactive images and examples to increase engagement and interactivity. Figures 2 and 3 provide an example of the low-fidelity prototypes.

### Data Collection

Study participants received the low-fidelity prototypes (ie, videos) via text message, WhatsApp Messenger, or email, depending on their preference, and were asked to watch the video at home on their own time prior to the scheduled qualitative interview. A bilingual or bicultural staff member trained in qualitative research techniques conducted interviews from January to August 2022. These interviews were held virtually, and they took place in either Spanish or English based on the participant’s choice. Each interview spanned 30 to 60 minutes in length and was audio recorded and transcribed verbatim by a professional service.

The staff member used semistructured interview guides. For the first 5 interviews, the interview guide asked general questions pertaining to likes and dislikes, queries about the program’s dementia content, examination of the ABC problem-solving strategy, assessment of the provided caregiving example, and a series of questions delving into video accessibility, design, and duration. For the subsequent 5 interviews, we revised the interview guide by adding questions focused on participant preferences, such as whether they preferred watching or listening to the video content, their favored method of receiving the videos, and their ideal video length. Finally, after another subsequent 5 interviews, we revised the interview guide again by incorporating additional inquiries centered around evaluating the images within the videos and improving viewer engagement.

### Data Analysis

We analyzed the transcripts in their original languages, either Spanish or English, using the qualitative data analysis techniques described by Saldaña [17]. We began by reading through all the transcripts and writing analytic memos to reflect on the content—noting insights, thoughts, and emerging patterns. We then applied deductive codes from a pre-established codebook to the interview transcripts (“protocol coding” [17]). The pre-established codebook was used in a prior qualitative study of STAR-C and was composed of codes representing components of the cultural treatment adaptation framework [14,18]. As an illustration, the codebook contained codes like “Materials and Semantics,” “Cultural Examples and Themes,” and “Therapeutic Framework” to capture cultural adaptations that were needed in the delivery of the intervention. In the next step, we applied inductive second-order codes to capture the details of participants’ feedback (“subcoding” [17]). For example, one of the inductive subcodes under “Materials and Semantics” was “Different BPSD examples” since multiple participants had suggested including in the intervention materials problem-solving examples for different BPSD. Afterward, we grouped the deductive and inductive codes into a smaller number of candidate themes that represented opportunities to improve the cultural adaptations of STAR-C (“pattern coding” [17]). We examined the interrelationship across and within the candidate themes and made refinements to ensure that excerpts within themes cohered and that each final theme was distinct from the others. Finally, we developed a statement to describe each of themes, which are presented in the following section. To manage this coding process, we used Dedoose (version 8.1.8; SocioCultural Research Consultants, LLC).

## Results

### Overview

Table 1 provides the characteristics of Latino caregivers who participated in this study and a description of their caregiving situation. The average age of caregivers was 48.9 (SD 11.1) years, 13 (87%) identified as women, 7 (47%) were the adult children of the people living with dementia, and 12 (80%) provided 35 hours or more of care per week.

**Table 1:** Characteristics of family caregivers and description of the caregiving situation.

Characteristics			Family caregivers (N=15), n (%)
Age (years), mean (SD)			48.9 (11.1)
Mexican, Mexican American, or Chicano			15 (100)
**Gender**			
	Woman	13 (87)	
	Man	2 (13)	
**Occupational status**			
	Retired	1 (7)	
	Employed	5 (33)	
	Unemployed	1 (7)	
	Homemaker	5 (33)	
	Other	3 (20)	
**Highest level of educational attainment, mean (SD)**			
	Less than high school	4 (27)	
	High school	5 (33)	
	Vocational or technical training	2 (13)	
	Some college	1 (7)	
	College graduate	2 (13)	
	Postgraduate	1 (7)	
**Devices owned**			
	Smartphone	14 (93)	
	Tablet	5 (33)	
	Laptop	7 (47)	
	Computer	6 (40)	
	Owns any device	15 (100)	
**Caregiver’s relationship to person living with dementia, mean (SD)**			
	Adult child (eg, daughter)	7 (46.7)	
	Spouse or partner	5 (33.3)	
	Other relative	3 (20)	
**Care provided per week (hours)**			
	35 or more	12 (80)	
	5-14	3 (20)	
Number of years providing care, mean (SD)			3.7 (2.4)
Caregiver and person living with dementia living together, mean (SD)			10 (66.7)

The qualitative analysis revealed adaptations that were needed in the (1) delivery of the STAR-C virtual intervention, (2) “dementia education” core component, (3) “ABC Problem-Solving” core component, and (4) “caregiver support strategies” core component. The sections below describe the need for these adaptations from the perspective of caregivers, as well as the changes that were made to the STAR-C web-based training modules in response to caregiver feedback.

### Theme 1: Adaptations to the Delivery of the STAR-C Virtual Intervention

#### Design Videos to be Accessible via Audio Only

Latino caregivers reported that they liked that the STAR-C videos combined visuals with narration. They reported that the narration helped to reinforce the visual content and vice versa. They said this combination of visuals and narration was engaging because it helped to hold their attention and made it easier for them to learn the content. For example, 1 Spanish-speaking caregiver said:

The thing is that audio and seeing the image are also easier in this type of application. It became more practical and easier for me to learn and understand compared to just reading and seeing it without any—well, at least in my opinion, my brain doesn't work the same way, I believe, but I liked the idea that you can see it, like imagine it, and create that idea.

However, while the ideal would be to watch the STAR-C videos, some caregivers mentioned that this may not always be practical. They said that they were often occupied with errands, household, and caregiving work, and that it would be nice in these circumstances to be able to listen to the video. For that reason, the caregivers suggested that the videos be designed to be accessible via audio only. Further, 1 Spanish-speaking caregiver stated:

Since sometimes one doesn't have much time to sit down and take the time to be looking at the screen. So, I put everything in the background while I do my daily tasks.

In response to caregivers’ feedback, we modified the narration script to ensure that it was independent of visuals and accessible via audio only. The narration focused on providing clear and descriptive explanations of the visual content. Instead of relying on visuals to convey information, the narration script described the key elements, actions, and visuals present in the video. This approach would enable caregivers to form a mental image and grasp the content without needing to see the visuals.

#### Make Videos Accessible on Multiple Platforms

Latino caregivers expressed a preference for having various methods of accessing the STAR-C videos, including phone applications such as Facebook Messenger, WhatsApp Messenger, YouTube, and other platforms. Caregivers desired video access through platforms they used regularly and were easily accessible on their preferred devices. For example, 1 Spanish-speaking caregiver stated:

No, but I hardly use my email. I do check my email, but almost all the information I receive, I receive it through WhatsApp. And then, I can see it right away without any problem.

A few caregivers encountered technical difficulties when attempting to access the videos such as lack of Wi-Fi access in their homes or limited storage on their phones, requiring troubleshooting. For example, 1 caregiver said she could not view the videos initially because her phone’s storage was full. Once the caregiver emptied her phone’s storage, she was able to view the videos. In addition, while the caregiver could view the videos on her computer, she did not have Wi-Fi at home and would need to connect the computer to her phone’s hotspot.

In response to caregivers’ feedback, we considered various options to accommodate preferences and technology access, to ensure optimal accessibility to the STAR-C videos. We would offer caregivers the option to receive the STAR-C videos on phone applications like Facebook Messenger, WhatsApp Messenger, and YouTube, as these are commonly used platforms for communication and information exchange. We would also optimize the video formats to reduce file sizes. Finally, we would offer troubleshooting support to assist caregivers who may face technical challenges while trying to view the videos.

#### Enable Caregivers to Easily Share Videos With Family Members

According to caregivers, the content in the STAR-C videos had a positive impact on communication and information sharing among caregivers and other family members who assist with caregiving. For that reason, caregivers expressed a strong desire to share the video content with their extended family, including siblings and other relatives involved in caregiving responsibilities. Sharing the videos empowered caregivers to improve their caregiving practices. For example, 1 Spanish-speaking caregiver explained:

Moreover, right away, I allowed myself—I don’t know if it was allowed, but I sent that information to my sisters-in-law and they were like, “Wow, wait, so I’m going to treat my dad like this, so I can stop this. So, I got irritated by this.” My sisters said, “I got it now,” because we truly are alone, there really isn’t much of a tool at hand that leads you to something like this, and even though it might seem small, a few minutes of video, it was truly very good. So, I can’t imagine everything that’s going to come [in the future with STAR-C]. Really, I appreciate it, truly.

Another Spanish-speaking caregiver highlighted the need for education and support at the individual level. They indicated that the program’s benefits extended beyond their immediate family, potentially benefiting other caregivers in similar situations. The caregiver explained:

I believe what we were missing was educating ourselves more about this… It could be the entire family, because it was in Mexico where my mom, the rest of my siblings, and I were. We were all taking care of dad, and sometimes, even then, we couldn’t manage. We were like five adults and my dad.

In response to caregivers’ feedback, we decided that it would be appropriate to encourage Latino caregivers participating in the research study to share the STAR-C videos with other family members if they wished, regardless of whether the other family members were also enrolled in the research study. In addition, we decided to welcome other family members within the same family to enroll as study participants if they were interested and met the eligibility criteria. The program’s content could serve as a catalyst for discussion, knowledge exchange, and support among primary caregivers and their extended network of caregivers.

### Theme 2: Adaptations to the “Dementia Education” Core Component

#### Expand Content to Improve Caregiver Understanding of Dementia

Latino family caregivers expressed the need for expanded content in the “Dementia Education” core component to enhance their understanding of dementia. They highlighted the importance of delving deeper into the nature of the disease, its progression, and the various stages it entails.

[The video] was very practical, the basics, what [dementia] is, the essentials, but at the beginning, yes, to know a little more about what that type of illness it is, like videos on how it forms in the brain and the stages, that it goes deeper because it progresses in stages and increases day by day. That's what I think.

Several caregivers identified common misconceptions about Alzheimer disease and memory loss in the Latino community, expressing surprise at the late stages of dementia when physical limitations manifest. They stressed the necessity of detailed information to comprehend the evolving challenges faced by people living with dementia. Caregivers emphasized the significance of incorporating this expanded content to enhance their knowledge and foster a more holistic understanding of dementia.

But that part surprised me a lot, which is that the body itself forgets its needs. And it's the final stage. And I would like it if they did include the stages of Alzheimer's or dementia. Because for most people, if you tell them, it's like “oh, they forgot things or put something in the wrong place or they get lost.” But the final stage is the one that very few people know about, it's what happens when your body, even if it gives signals, you don't recognize them. It's like a baby who can't tell you they’re thirsty, they’re hungry, their stomach hurts, and things like that. So, that's when it gets complicated and obviously, the end is near.

In response to caregivers’ feedback, we further expanded the content of the first video focused on teaching caregivers about ADRD. Our goal was to provide a comprehensive understanding of dementia, untangling its root causes and distinguishing it from the natural aging process. We describe the different stages of dementia to offer Latino caregivers a better understanding about types of cognitive, emotional, and behavioral changes to expect in their family member with dementia as the disease progresses.

#### Encourage Caregivers to Empathize With Person Living With Dementia

Latino caregivers expressed the importance of fostering empathy toward individuals living with dementia. They highlighted the need to understand and acknowledge the unique challenges faced by both the caregivers and the person with dementia.

A Spanish-speaking caregiver found the examples provided in the program to be highly relatable, acknowledging the frustrations experienced by both themselves and their patients. They said:

I loved the examples they provided because they're very real, of diagnosing changes for oneself, the patient or client, or the family. Why? Because it's not the same; it's frustrating for them, as much as for us, because they don't understand us, and we don't understand them. So, we can't say that one is normal, because, to be honest, none of us are normal [laughter]. But yes, we can understand that diagnosis, put ourselves in the shoes of that person, or think about how I would feel if I see that the person taking care of me is frustrated by something I don't even know about, or I'm not comprehending.

Caregivers also expressed the need to put themselves in the shoes of the person with dementia. Further, 1 Spanish-speaking caregiver said:

The only thing is that it would also be good to mention how it's frustrating for them and for us. We should put ourselves in their shoes, how would we like to be treated if we were the ones sick, how would we want to be treated? In terms of frustration or reactions... They won't react the way we would want them to or how we would like them to react, because they have a condition, we don't.

In response to caregivers’ feedback, we integrated content into the STAR-C videos that would remind caregivers to be empathetic and to consider the perspective of people living with dementia. The added content emphasizes the importance of understanding the frustrations experienced by both caregivers and people living with dementia and the need to approach interactions with empathy and compassion.

#### Provide Education on how to Reduce Risk of Developing Dementia

Caregivers indicated the need for comprehensive education on reducing their own risk of developing dementia. They highlighted the importance of understanding the impact of dementia while also learning about strategies to combat it. A Spanish-speaking caregiver said:

The thing is, for example, I'm looking at my husband's case and I think about myself, and I think, “Well, what can I do to prevent what happened to my husband from happening to me?” Because what will happen if I develop dementia? What will happen to both of us? So, I would like to have more information. What can I do to avoid this? Because I am his caregiver.

Another Spanish-speaking caregiver, after learning about the prevalence of dementia among Hispanics in the STAR-C video, expressed the need for more information on prevention and early signs. They said:

That's really good [the information in the module]. I was genuinely surprised when it said that one in every eight Hispanics has or will develop, right? So, it's a bit alarming, and I think, wow, I don't know, I would like to know more about whether there would be any way to prevent it. It would be great to have more information about prevention or the signs—as it says there, some forgetfulness is normal, certain forgetfulness, right? Like now, being busy with a thousand things, I forgot, and believe me, it happens to me, but I know it's because I have a lot on my plate and I try to do them all. But I would really like if there was information about whether there's any way to prevent this condition.

Caregivers’ feedback confirmed our previous findings about the need to modify the STAR-C content to include comprehensive education on dementia. In response to caregivers’ feedback, we will also be providing caregivers with information on reducing their own risk of developing the condition. By providing caregivers with guidance and knowledge on prevention strategies, STAR-C can empower them to take proactive steps in safeguarding their cognitive health and that of their loved ones.

### Theme 3: Adaptations to the “ABC Problem-Solving” Core Component

#### Simplify the “ABC” Problem-Solving Acronym in Spanish

The “ABC” problem-solving approach in the STAR-C program was regarded as helpful by some Latino caregivers, who found the provided examples to be effective and relatable. However, it was acknowledged that understanding the acronym (“Activators, Behaviors, Consequences”) could be a bit challenging for others. Further, 1 Spanish-speaking caregiver explained why she liked the ABC problem-solving approach:

So, I found the video to be very original, very realistic. It was done very well because I felt identified. The three ways they presented it, in A, B, and C, personally, it felt very real to me, I loved it, almost perfect, because these are situations that do happen and changes that we do need to make. From the beginning, we don't know how to do it, but with this video or the app that they're going to develop, it seems very practical to me because it will provide a lot of tools and strategies to people who have no idea how to go about it. Like us in the beginning, we were learning as we went through each day.

Another Spanish-speaking caregiver initially faced challenges in understanding the ABC acronym but gained clarity once the video explained it further.

Yes, some of those words were a bit difficult for me to understand, but later on, it was explained what each of them meant.

Further, 1 Spanish-speaking caregiver reported that while they could understand the content, including the ABC acronym, well due to their extensive Spanish language skills, they acknowledged that a person with limited education might struggle to understand it clearly.

I can understand it perfectly, but I think I can understand because I have a very good Spanish. I was a Spanish teacher for many years, so my language and vocabulary are quite extensive. However, I believe that if the same video had to be heard by someone with limited education, they probably wouldn't understand it, at least not clearly.

These quotes highlight the caregivers’ perspective on the need to simplify the “ABC” problem-solving acronym in Spanish. While some caregivers found the explanations of each letter in the acronym helpful, others reported that individuals with limited education may potentially face challenges in understanding the acronym. In response to caregivers’ feedback, we simplified the ABC acronym to enhance comprehension and accessibility for a wider range of caregivers. Initially, the ABC acronym was translated as A for “activadores” (activators), B for “comportamiento” (behavior), and C for “consecuencias” (consequences). However, based on the feedback received, we took an additional step to simplify it entirely in Spanish, resulting in the revised form as “las 3 Cs” (the 3 Cs) representing C for “causas” (causes), C for “comportamiento” (behavior), and C for “consecuencias” (consequences).

#### Add More Problem-Solving Examples With Different BPSDs

Caregivers conveyed a strong desire for the STAR-C program to incorporate a greater variety of problem-solving examples that cover different BPSDs. While some caregivers appreciated the existing examples, they emphasized the importance of including a more extensive range of stories and behaviors to address the diverse challenges encountered in dementia care. For example, 1 Spanish-speaking caregiver stated:

I liked everything, the only thing is that I would like them to add a bit more different stories, with different behaviors.

Another Spanish-speaking caregiver shared their personal experience with their mother’s behavior and the importance of addressing such situations.

Yes, my mom experiences a lot of panic episodes, and I didn't see that in the video, so in my own way, I handled panic situations in her illness, like waking up at night in a panic: “Where am I? Who am I? Where are we?” So, I didn't see in the video strong things like that, like screaming, situations where you don't know what to do as a family member, so you just hug them. I hugged my mom, I hugged her, I hugged her, and I said, “Calm down, we're okay. I'm your daughter, we're here.” I mentioned the house, the surroundings, everything. But she has those night panics out of fear very often.

These quotes underscore the caregivers’ interest in having a more extensive selection of problem-solving examples that address various BPSDs, such as aggression and panic attacks. In response to caregiver’s feedback, we will include a broader range of scenarios and behaviors, so that caregivers can gain invaluable insights and strategies to effectively manage the diverse challenges associated with BPSDs.

#### Demonstrate Problem-Solving With Real People

Latino caregivers reported a desire for more realistic and relatable problem-solving examples in the STAR-C program. They suggested incorporating videos featuring real interactions between caregivers and persons living with dementia to enhance the learning experience. Further, 1 Spanish-speaking caregiver explained:

Perhaps, in the example—I mean, I don't know how much of the video or program is left [to be developed], but maybe, I don't know, perhaps the depiction of two people acting out the situation, maybe it would look much more professional or more—you know, I understand that this requires investment and it requires many things.

Another Spanish-speaking caregiver reported interest in observing caregiving interactions to better understand effective communication strategies.

Perhaps some of this information would be clearer in a video format or as an interaction between two people. Yes, specifically with an Alzheimer's patient. Because if they're [the program] going to be incorporating strategies on how to talk, how to interact, it would be very useful to see the behavior live; how it's happening, how one can communicate with that person.

Incorporating videos of real people and interactive caregiving scenarios in the STAR-C program can provide caregivers with tangible and relatable examples of problem-solving techniques. Based on caregivers’ feedback, future adaptations of STAR-C should demonstrate the app of the problem-solving techniques with real people. These demonstrations may enhance caregivers’ experience with the program by presenting solutions in a dynamic and engaging manner.

### Theme 4: Adaptations to the “Caregiver Support Strategies” Core Component

Latino caregivers emphasized the importance of seeking assistance and support to alleviate the burdens of caregiving. Many highlighted the value of reaching out to family members or friends when feeling fatigued or overwhelmed, recognizing the need for rest and self-care. A Spanish-speaking caregiver described how the STAR-C program needed to encourage caregivers to take care of their own health and well-being. The caregiver said:

[For caregivers] to be well-rested. That's why I mention seeking help in some way, even from family or friends, you can say “I need—” when you feel tired or stressed, very stressed, you need to be able to call someone and say, “Can you take care of my mom for a while? Can you take care of my dad or my wife? I need a break.” Because if you're not well-rested or already feeling overwhelmed—I say this because, for example, my sister, when she was taking care of my mom, my sister wasn't emotionally well. She had her own problems, so when it was her turn to care for my mom, there were almost always issues because she didn't have enough patience.

Participants collectively acknowledged the importance of supporting caregivers themselves, not just the care recipients. They emphasized the need for caregivers to take regular breaks and engage in activities that promote mental well-being. For example, a Spanish-speaking caregiver stated:

I just wanted to mention that also the people-- sometimes the people who care for others also need to be taken care of, to take a break or do something different in order to continue, to be mentally well enough to keep taking care of our loved ones.

This comment highlights the importance of recognizing the caregiver’s mental and emotional health as it directly impacts their ability to provide effective care. In response to caregivers’ feedback, we integrated content throughout the various modules (not just the last module) about the importance of caregiver health and well-being. The content emphasizes the need for caregiver rest, support, and self-care, so that caregivers can be better equipped to provide optimal care to their loved ones.

## Discussion

### Principal Results

This study’s objective was to test and refine the preliminary cultural adaptations to the STAR-C web-based training modules for Latino caregivers. Our qualitative analysis identified key adaptations required in (1) the delivery of STAR-C, (2) the “dementia education” core component, (3) the “ABC problem-solving” core component, and (4) the “caregiver support strategies” core component. Caregivers expressed a desire for STAR-C videos to be accessible through audio-only formats, and they highlighted the importance of making the videos available on various platforms, including those commonly used in their community. They also emphasized the need for the videos to be easily shareable with family members to enhance communication and caregiving practices. In response, we tailored the narration script to facilitate audio-only access and optimized video formats for widespread accessibility. Moreover, caregivers requested comprehensive education on dementia prevention and understanding the disease’s stages, advocating for expanded content within the “Dementia Education” core component. This resulted in additional content aimed at enhancing caregivers’ understanding of dementia progression and challenges. To further foster empathy, caregivers suggested highlighting the importance of viewing the world from the perspective of individuals living with dementia. Consequently, we incorporated elements emphasizing empathy into the program. Caregivers also sought to incorporate strategies for reducing the risk of developing dementia, leading to the inclusion of content addressing preventive measures. Within the “ABC Problem-Solving” core component, caregivers expressed a need to simplify the “ABC” acronym in Spanish and expand problem-solving examples. We responded by simplifying the acronym and incorporating more problem-solving scenarios to cater to diverse challenges. Additionally, caregivers desired realistic problem-solving examples featuring real interactions between caregivers and persons with dementia. Lastly, caregivers emphasized the importance of caregiver well-being, prompting us to underscore self-care, rest, and seeking support to ensure caregivers’ mental and emotional health is prioritized. These adaptations collectively refine the STAR-C web-based training modules for Latino caregivers and provide a culturally tailored, evidence-based intervention to support dementia caregiving in this community.

### Comparison With Prior Work

Our study identified several key adaptations needed to enhance the cultural relevance and effectiveness of the STAR-C web-based training modules for Latino caregivers. Notably, caregivers expressed a strong desire for videos to be accessible through audio-only formats, a finding that aligns with a study conducted among Hispanic participants from Spain [19], where a similar preference for audio accessibility was observed. The importance of making videos available on various platforms, as highlighted in our study, is also consistent with research where caregivers expressed openness to learning caregiving information from diverse sources and settings [20].

The cultural adaptations identified in this study to enhance the relevance and effectiveness of the STAR-C web-based training modules for Latino caregivers may have applicability to caregivers from other racial and ethnic minority groups as well. For example, collectivist and familism values tend to be strong in Asian cultures that emphasize family and community interdependence [21,22]. As such, making the STAR-C videos easily shareable with family members to facilitate communication and coordinated caregiving practices may also resonate with Asian caregivers.

Furthermore, our study aligns with prior research by emphasizing the need for comprehensive education on dementia prevention and understanding the disease’s stages [20,23]. In a study conducted among various ethnic groups, including Hispanic or Latinos, African Americans, and Asian Americans, it was found that a common lack of knowledge about the early signs of Alzheimer disease existed [23]. This knowledge gap underscores the importance for comprehensive dementia education, a point that resonates with our study’s findings. Additionally, another study similarly stressed the importance of greater education regarding the diversity and spectrum of dementia-related symptoms [20].

Caregivers also strongly advocated for simplifying the ABC acronym in Spanish, adding more problem-solving examples, and demonstrating problem-solving with real individuals. These adaptations align with a study assessing Spanish language health information via videos [24], which found that participants preferred videos featuring increased actor participation, as it enhanced their ability to relate the content to real-life situations.

### Limitations

Our study has limitations worth noting. First, the low-fidelity prototypes of the STAR-C web-based training modules (ie, videos) we used in this study lacked the realistic appearance and comprehensiveness of the full virtual STAR-C program. This may have limited our ability to obtain accurate perceptions and feedback, as caregivers might not have fully understood or engaged with the low-fidelity prototypes as they would have with a higher-fidelity version of the full STAR-C program. As a next step in this research, we plan to pilot test high-fidelity prototypes of the STAR-C web-based training modules. Second, caregivers’ feedback about STAR-C is influenced by their personal preferences and experiences, which may result in us inadvertently prioritizing features or solutions that appeal to the specific subgroup of Latino caregivers participating in this study rather than broader populations of Latino caregivers. We tried to address this limitation by interviewing a diverse group of caregivers including both male and female caregivers (most Latino caregivers tend to be women), caregivers of different ages and family roles (ie, spouse vs child caregiver), and caregivers with different educational backgrounds.

### Conclusions

This study addresses a critical gap in the field of dementia caregiving, particularly for Latino caregivers who face unique challenges and disparities in health outcomes. The culturally adapted version of the virtual STAR-C program represents a significant step forward in bridging this gap. Our findings highlight the importance of tailoring interventions to meet the specific needs of Latino caregivers, considering cultural values and beliefs that shape caregiving interactions and experiences. The key adaptations we needed to make to the STAR-C web-based training modules, including accessibility improvements, expanded dementia education, enhanced problem-solving strategies, and a focus on caregiver well-being, underscore the importance of cultural adaptation of evidence-based caregiver interventions for Latino families. While further research is needed to assess the efficacy of these adaptations, we believe that the virtual STAR-C intervention has the potential to improve health care services and health outcomes for Latino people living with dementia and their family caregivers. By addressing the pressing need for culturally adapted evidence-based interventions, we aim to promote more favorable experiences with the intervention and ultimately reduce the health disparities associated with dementia caregiving in this community. This work contributes to the broader mission of advancing health care services for older adults by using technological innovations, serving the interest of health professionals and family caregivers of older adults.

## Abbreviations

ABC: Activators-Behaviors-Consequences
ADRD: Alzheimer disease and related dementias
BPSD: behavioral and psychological symptoms of dementia
REDCap: Research Electronic Data Capture
STAR-C: STAR caregiver
